# A Multi-Site Evaluation of Innovative Approaches to Increase Tuberculosis Case Notification: Summary Results

**DOI:** 10.1371/journal.pone.0094465

**Published:** 2014-04-10

**Authors:** Jacob Creswell, Suvanand Sahu, Lucie Blok, Mirjam I. Bakker, Robert Stevens, Lucica Ditiu

**Affiliations:** 1 Stop TB Partnership, Geneva, Switzerland; 2 Royal Tropical Institute (KIT), Health, Amsterdam, The Netherlands; 3 Royal Tropical Institute (KIT) Biomedical Research, Amsterdam, Netherlands; 4 HLSP, London, England; National Institute of Infectious Diseases, Japan

## Abstract

**Background:**

Globally, TB notifications have stagnated since 2007, and sputum smear positive notifications have been declining despite policies to improve case detection. We evaluate results of 28 interventions focused on improving TB case detection.

**Methods:**

We measured additional sputum smear positive cases treated, defined as the intervention area's increase in case notification during the project compared to the previous year. Projects were encouraged to select control areas and collect historical notification data. We used time series negative binomial regression for over-dispersed cross-sectional data accounting for fixed and random effects to test the individual projects' effects on TB notification while controlling for trend and control populations.

**Results:**

Twenty-eight projects, 19 with control populations, completed at least four quarters of case finding activities, covering a population of 89.2 million. Among all projects sputum smear positive (SS+) TB notifications increased 24.9% and annualized notification rates increased from 69.1 to 86.2/100,000 (p = 0.0209) during interventions. Among the 19 projects with control populations, SS+TB case notifications increased 36.9% increase while in the control populations a 3.6% decrease was observed. Fourteen (74%) of the 19 projects' SS+TB notification rates in intervention areas increased from the baseline to intervention period when controlling for historical trends and notifications in control areas.

**Conclusions:**

Interventions were associated with large increases in TB notifications across many settings, using an array of interventions. Many people with TB are not reached using current approaches. Different methods and interventions tailored to local realities are urgently needed.

## Introduction

In the early 1990s, the World Health Organization (WHO) launched DOTS as a strategy incorporating the fundamentals for tuberculosis (TB) control with targets for TB case detection and treatment success [1]. Through the 1990s and into the 2000s DOTS was expanded rapidly driven by the main targets of detecting and notifying 70% of estimated incident sputum smear positive (SS+) TB cases and achieving 85% treatment success [2]. From 1991 until 2008 the gains were impressive: SS+TB case notification increased from 11% to 64% of the estimated incident cases, mainly through passive case finding at public facilities [3]. However, since 2008 all forms notifications have stagnated and 3 million incident TB cases (34% of current global estimate) are still either not detected or not notified, with only half of the 12 million prevalent cases of undiagnosed TB likely to be detected during a year [4]. Most undetected/un-notified all forms incident cases are in south-east Asia (1.2 million) and Africa (0.8 million), with the poor and most vulnerable suffering disproportionately from deficient access to TB services and bearing most of the overall burden [5]. The TB community has produced policies to improve case detection [6]–[8] and move towards the goal of universal access and 100% case detection [9]. While passive facility-based case finding (the updated DOTS component now being part of a broader Stop TB Strategy) is still essential for patient management, it may not be able to penetrate communities well enough to make a rapid impact on the epidemic [5], [10].

Passive case finding is limited by slow initiation of health seeking in people with TB who can be minimally symptomatic [11], compounded by barriers to access care (cultural, geographical and financial), poor diagnostic services, and insensitive screening algorithms [5], [9].

Two distinct initiatives have been launched to stimulate and gather evidence for action: FIDELIS (2003–2007) and TB REACH (2010–present). FIDELIS interventions covered a time when case notifications were rapidly increasing globally and were heavily focused on expansion of national DOTS programmes. China and Pakistan alone accounted for 74% of all gains in case notifications respectively under FIDELIS [12]. In 2010 the Canadian International Development Agency (CIDA) provided funding for TB REACH, administered by the Stop TB Partnership. Through a competitive selection process, one year grants were provided to institutions and organizations proposing to increase case finding and then scale up contingent on other funding [13]. We present findings of an evaluation of the first wave projects.

## Methods

After its inception in January 2010, TB REACH launched a call for proposals and a group of projects selected by an independent proposal review committee was awarded funding in May 2010. One year grants up to 1,000,000 USD were given to institutions and organizations that focused on increasing the number of SS+ cases detected and treated. Projects were selected based on feasibility, innovation, targeting of populations with limited access to care, the numbers of additional SS+TB cases they proposed to find, and estimated cost. Multiple proposals from the same country were encouraged, and applicants had to present a letter of support from the National TB Programme (NTP) to help ensure treatment would be available for additional cases found and to guarantee sharing of notification data. Applicants were requested to try new strategies, or introduce an approach that had been proven effective elsewhere, and to focus on targeting and filling gaps, rather than on general improvements to the existing system. Thirty projects covering 19 different countries were selected from 192 applications with 18.4 million USD awarded. Initial activities generally began in the 4^th^ quarter of 2010 although projects had different start dates for case finding activities.

From October 2010 until March 2012, 29 projects completed at least 4 quarters of case finding activities. This number excludes a project in Burkina Faso which did not begin activities until late 2011 due to administrative problems, and was not included in the analysis. Additionally, we were unable to collect and verify the routine NTP data of the project in Yemen due to civil unrest, leaving a total of 28 projects for this analysis. Eleven of the 28 projects were headed by international NGOs, eight by National/State/Local TB Control Programmes, six by domestic NGOs, two by academic institutions, and one by the International Organization for Migration. The projects covered a total population of 89.2 million (evaluation population). Case finding interventions were carried out for 123 cumulative quarters. The total financial expenditure of the 28 projects during the reporting period was 14.9 million USD. General project characteristics are displayed in Table 1.

**Table 1:** Overview of TB REACH Wave 1 Projects.

Country/Project	Total Budget USD	Quarters of TB Case Finding Activities	Budget Spent USD	Population: Evaluation Area	Population: Control Area
Afghanistan NTP	626,796	5	618,785	9,838,000	207,499
Afghanistan ATA	541,346	4	541,346	4,399,997	387,251
DRC Katanga	538,108	5	459,306	3,306,667	3,078,498
DRC Equateur	964,673	5	835,091	5,134,800	3,534,839
DRC Kasai	604,928	5	516,778	3,311,829	3,624,724
DRC CRS	870,930	4	870,930	3,178,000	886,475
Ethiopia LSTM	689,163	5	689,163	3,053,083	3,141,622
Ethiopia IA	156,490	4	156,490	855,789	1,689,455
Laos IOM	297,460	4	288,824	1,601,398	1,400,000
Laos PSI	468,308	5	402,389	3,659,541	731,401
Lesotho FIND	379,788	4	379,788	720,109	1,159,891
Nepal FHI	772,035	4	714,040	4,673,517	262,542
Nigeria CRS	1,000,000	6	649,117	3,693,283	353,844
Pakistan NTP	937,023	4	655,232	6,045,105	4,059,282
Pakistan IND	511,199	4	511,199	1,785,000	1,204,000
Rwanda WVC	315,000	5	285,829	1,364,340	1,100,771
Tanzania NIMR	509,355	4	505,097	977,626	1,524,632
Uganda BRAC	231,047	4	198,370	2,251,500	541,800
Uganda AMREF	857,554	5	580,036	1,918,400	172,100
Benin NTP	524,441	4	508,932	8,034,522	NA
Kenya IMC	966,780	4	966,780	1,767,952	NA
Kenya KAPTLD	994,806	5	994,806	6,000,000	NA
Pakistan BC	151,150	4	151,150	22,730	NA
Pakistan PP	500,000	4	249,747	200,000	NA
Somalia WVC	760,000	4	336,118	5,655,000	NA
Sudan EPILAB	746,673	4	557,256	4,162,908	NA
Zambia CRDRZ	1,000,000	4	843,505	11,000	NA
Zimbabwe CHD	507,635	4	455,965	1,542,534	NA
Burkina Faso NTP*	445,758				
Yemen LSTM†	287,621				
**Total**	**18,156,067**	**123**	**14,922,069**	**89,164,630**	**29,060,626**

*Project started project activities in Q4 2011 and was not included in the analysis.

† M&E team were unable to verify project and NTP data and was excluded from the analysis.

An independent monitoring and evaluation (M&E) team reviewed and validated all project data. Each project defined their target population (the group(s) of people at which the interventions were directly aimed that is a subset of the evaluation population) and formulated their evaluation population. The evaluation population was usually one or more NTP basic management units (BMU) or sites to which members of the target population would normally present for diagnosis and treatment, and so tended to include non-target populations too. The main outcome of interest was the number of *additional* SS+ cases treated, defined as the increase in TB case notification from NTP treatment registers within the reporting area (i.e. evaluation population) during the intervention period compared to the same area's notifications from the previous year. We collected data on all forms of TB (total cases notified) cases as well for the purpose of project evaluation.

Control populations were selected in consultation with the M&E team to be as comparable as possible to the evaluation populations and to have sufficient geographical separation to minimize any spillover effect from or into the evaluation population. Population estimates were obtained for 2010 using data provided by the NTP or national bureau of statistics.

In order to allow accurate projections and to control for trend, quarterly historical case notification data were collected from both control and evaluation populations for the three years prior to the interventions. Projects reported case notifications using standardized quarterly forms and official NTP notification data, project-specific screening and testing indicators, any potential external factors influencing case finding in the evaluation and control areas such as drug stock outs or political instability, information on data quality, and financial expenditures. Projects received at least one M&E field visit during the implementation period to address technical issues, validate reported information, and help improve data quality through reviews of NTP registers. Routinely collected quarterly NTP data was used with no personal identifiers for this analysis, so ethical approval was not required.

### Statistical analysis

We used several approaches to measure the projects' impact on TB notifications. Additional cases of SS+ and all forms of TB were calculated from the difference between case notifications during the project implementation period and notifications from the corresponding number of quarters from the previous year (historical baseline). If a project had five quarters of implementation during the evaluation period, the one-year historical baseline was multiplied by a factor 1.25 unless a strong seasonal trend in notification was observed, in which case the corresponding historical quarter was multiplied by two. In Nepal, four-month reporting periods were converted to quarterly data to conform to other project reporting. To generate an estimate of the expected cases in each population we used simple linear regression to fit a trend line through the historical notifications assuming historical trends continued and then compared them to observed notifications. In one project there was a strong degree of seasonality in the data so the trend line was adjusted on a quarterly basis dependent on the rate of change from the previous year's corresponding quarter instead of using the linear model.

For individual projects we compared the mean SS+ notification rates per 100,000 population between baseline and intervention periods using the Kruskal Wallis one-way ANOVA for non-parametric data. Population data were held constant throughout the baseline and intervention periods. To compare quarterly notification rates observed during baseline and intervention periods across all projects, we weighted each project based on its proportional population size. For the 19 projects that had control populations we calculated individual notification rate ratios using negative binomial regression for over-dispersed cross-sectional TB notification data, accounting for both fixed and random effects. We used an offset based on the population in the evaluation and control populations. The 9 projects that had no control population were excluded from the analysis, as control population data was an integral reference. To compare the change in quarterly notification rates by case finding activity, we ran Mann-Whitney tests for non-parametric data. Statistical analyses were performed using Stata/IC version 11.

## Results

Almost all projects implemented more than one case finding intervention. Community volunteers, paid or unpaid, were part of 14 (50%) of projects. Six projects (21%) included private sector providers. In 15 projects (54%), mobile case finding activities were performed outside health facilities in the form of mobile diagnostic teams or by chest camps. Improved diagnostics including LED microscopes, Xpert MTB/RIF (Cepheid, Sunnyvale, CA, USA), digital x-ray, laboratory upgrades and frontloaded sputum collection were employed in nine projects (32%). Most projects targeted one or more population groups at high risk of developing TB. These included: contacts of people with TB (20 projects, 71%), migrants, internally displaced persons, miners, people with HIV, prisoners, and people with difficultly in accessing diagnosis and treatment such as rural and urban poor. A summary of project characteristics and intervention types can be found in Table 2. For a short description of each project's approach, see File S1.

**Table 2:** Summary of TB REACH Wave 1 Interventions.

Country/Project	Case Finding Strategies						Risk Groups Screened					
	Community Health Workers	Improved Diagnostics	Mobile Outreach	Sputum Transport	PPM	Demand Generation/ACSM	Contacts	Refugee/IDP/Migrants	Urban Slums	PLHIV	Prisons	Other*
Afghanistan NTP			1				1	1			1	
Afghanistan ATA			1				1	1			1	
Benin NTP		1	1			1						
DRC Katanga	1		1	1		1	1					1
DRC Equateur	1		1	1		1	1					1
DRC Kasai	1		1	1		1	1					1
DRC CRS	1		1		1	1	1				1	1
Ethiopia LSTM	1	1		1		1	1					
Ethiopia IA	1			1		1	1					
Kenya IMC	1		1	1		1	1		1			
Kenya KAPTLD	1		1		1	1	1		1	1		
Laos IOM	1			1		1		1				
Laos PSI					1	1	1			1		1
Lesotho FIND	1	1		1		1						
Nepal FHI			1				1	1		1	1	1
Nigeria CRS	1	1			1		1					1
Pakistan NTP		1	1		1	1						
Pakistan PP											1	
Pakistan BC		1					1					
Pakistan IND		1	1	1	1	1	1		1			
Rwanda WVC	1		1				1					1
Somalia WVC			1			1	1	1			1	1
Sudan EPILAB								1			1	1
Tanzania NIMR		1	1			1			1		1	
Uganda BRAC	1			1		1	1				1	
Uganda AMREF							1			1		
Zambia CRDRZ		1									1	
Zimbabwe CHD	1			1		1	1		1	1		
Burkina Faso PAMAC Yemen LSTM												
**Totals**	**14** (50%)	**9** (32%)	**15** (54%)	**11** (39%)	**6** (21%)	**18** (64%)	**20** (71%)	**6** (21%)	**5** (18%)	**5** (18%)	**10** (36%)	**10** (36%)

1 = yes

*Includes miners, military/police personnel, sex workers, drug users, women etc.

Percentages are based on 28 projects as Burkina Faso PAMAC and Yemen LSTM are excluded from analyses.

Pre-intervention, the NTPs reported 69,305 cases of SS+TB in the projects' evaluation population (Table 3), which increased to 86,541 (24.9% increase, 17,236 cases) in the intervention period. There was marked heterogeneity, with four projects reporting a net decrease in notified SS+TB cases compared to pre-intervention data. Among the 24 projects that reported gains, 17,686 additional cases were detected, representing a 32.1% increase from the baseline period. Among the 19 projects with control populations, much greater increases over baseline figures were reported for the intervention period (SS+TB case notifications increased from 40,832 to 55,908; a 36.9% increase) than control populations (28,820 to 27,788; a 3.6% decrease). Similar changes were noted for all forms of TB in the 28 evaluation populations (an overall increase over the baseline of 18,378 cases, with 22 of 28 intervention areas notifying additional cases).

**Table 3:** Summary of TB REACH Wave 1 Case Finding Results – Additional Cases and Trend Adjusted Estimates.

	Control Population						Evaluation Population					
Country/Project	Historic Cases		Intervention Period Cases		Additional SS+ Cases (% change)	Trend Adjusted SS+ Expected cases: (CI)	Historical Baseline Cases		Actual Intervention Period Cases		Additional SS+ Cases (% change)	Trend Adjusted SS+ Expected cases: (CI)
	SS+†	All Forms	SS+	All Forms			SS+	All Forms	SS+	All Forms		
												
Afghanistan NTP	225	928	406	786	181 (80.4%)	287 (227–346)	4351	7257	4777	8260	426 (9.8%)	4778 (4165–5391)
Afghanistan ATA	255	479	154	259	−101 (−39.6%)	294 (247–342)	1378	3412	2382	4087	1004 (72.9%)	1314 (1197–1432)
DRC Katanga	5349	8483	4831	7463	−518 (−9.7%)	5122 (4940–5305)	3673	5220	4802	6353	1130 (30.8%)	4198 (3984–4413)
DRC Equateur	4581	5773	3742	4951	−839 (−18.3%)	4600 (4376–4824)	3740	5058	5767	6773	2027 (54.2%)	4136 (3916–4356)
DRC Kasai	6919	8796	6313	8040	−606 (−8.9%)	7763 (7208–8319)	4028	4974	5145	6360	1117 (27.7%)	4575 (4328–4822)
DRC CRS	414	561	415	536	1 (0.2%)	402 (286–518)	1777	3479	2610	4023	833 (46.9%)	1790 (1670–1911)
Ethiopia LSTM	1186	2393	1370	3179	184(15.5%)	1221 (1122–1319)	2551	3980	5090	7071	2539 (99.5%)	2409 (2240–2578)
Ethiopia IA	754	1744	847	1774	93 (12.3%)	660 (546–775)	358	882	687	1202	329 (91.9%)	384 (340–428)
Laos IOM	666	813	760	930	94 (14.1%)	601 (540–663)	987	1147	1149	1344	162 (16.4%)	895 (811–979)
Laos PSI	338	411	390	467	52 (15.4%)	368 (334–402)	2089	2494	2179	2717	90 (4.3%)	2272 (2179–2366)
Lesotho FIND	1872	5169	1627	4548	−245 (−13.1%)	1836 (1689–1984)	1084	2943	1124	2793	40 (3.7%)	1145 (944–1346)
Nepal FHI	1935	4775	2093	4449	158 (8.2%)	2279 (2037–2520)	4373	7950	4338	7849	−35 (−0.8%)	4571 (4162–4979)
Nigeria CRS	216	343	167	227	−49 (−22.7%)	192 (148–236)	2184	3516	3038	4526	854 (39.1%)	2366 (2137–2595)
Pakistan NTP	2555	5225	2960	5663	405 (15.9%)	2578 (2366–2791)	2455	4881	5538	8648	3083 (125.6%)	2515 (2292–2738)
Pakistan IND	255	547	217	513	−38 (−14.9%)	262 (227–297)	771	1543	1292	3230	521 (67.6%)	861 (797–926)
Rwanda WVC	620	1104	613	942	−7 (−1.1%)	588 (489–687)	845	1316	805	1262	−40 (−4.7%)	895 (820–971)
Tanzania NIMR	110	239	89	240	−21 (−19.1%)	127 (112–142)	629	1539	885	1754	256 (40.7%)	649 (601–697)
Uganda BRAC	393	633	634	891	241(61.3%)	406 (367–446)	1779	3238	2259	4243	480 (27.0%)	1837 (1749–1924)
Uganda AMREF	178	380	160	345	−18 (−10.1%)	180 (161–199)	1781	2908	2041	3391	260 (14.6%)	1947 (1857–2037)
Benin NTP	NA						3178	3841	3593	4318	415 (13.1%)	3134 (3040–3230)
Kenya IMC	NA						3349	7412	3121	7493	−228 (−6.8%)	3545 (2738–4352)
Kenya KAPTLD	NA						12105	32893	12780	31819	675 (5.6%)	11613 (10994–12232)
Pakistan BC*	NA						34	74	518	564	484 (1423.5%)	
Pakistan PP	NA						106	166	343	565	237 (223.6%)	54 (6–101)
Somalia WVC	NA						1801	n/a	2253	n/a	452 (25.1%)	1718 (894–2543)
Sudan EPILAB	NA						5661	11474	5514	12091	−147 (−2.6%)	5071 (4861–5281)
Zambia CIDRZ	NA						38	185	165	373	127 (334.2%)	34 (22–47)
Zimbabwe CHD							2201	7148	2346	6197	145 (6.6%)	2417 (2302–2531)
Burkina Faso PAMAC	NA						NA					
Yemen LSTM	NA						NA					
**Totals**	**28820**	**48794**	**27788**	**46203**	**−1032**	**29767 (28113–31422)**	**69305**	**130929**	**86541**	**149306**	**17236**	**71124 (67289–74960)**

*Unable to generate trends due to lack of historical baseline data.

† Sputum Smear Positive abbreviated to SS+.

Based on historical trends, an expected 71,124 (95% CI: 67,289–74,960) SS+ cases would have been notified among 28 projects. Of all projects, 19 (68%) had observed SS+ counts during the intervention period which were above the 95% confidence interval for the expected count. The observed counts were within the confidence intervals for seven (25%) projects, one (3%) project's observed counts fell below the 95% confidence interval, and one project was excluded from analysis due to insufficient historical data.

Mean SS+TB notifications rates increased significantly in 17 (61%) of the 28 projects, including 14 (74%) of the 19 projects with control populations (Table 4). In the 19 control areas, notification rates dropped significantly in four (21%) and increased in two (10.5%). During the baseline period annualized notification rates in intervention areas were 69.1/100,000 for all 28 projects and 57.7/100,000 among the 19 with controls. During the intervention periods the annualized rates increased to 86.2/100,000 (p = 0.0209) for all projects and 79.0/100,000 (p = 0.0209), among 19 with control populations. There was no statistically significant difference in notification rates between the baseline and intervention period overall for the 19 control populations (85.6/100,000 to 83.2/100,000 p = 0.2482).

**Table 4:** Summary of TB REACH Wave 1 Case Finding Results – Quarterly Notification Rates.

	Control Population			Evaluation Population		
	Mean SS+ Notification Rate†			Mean SS+ Notification Rate		
Project	Historical	Intervention	P Value	Historical	Intervention	P Value
Afghanistan NTP	86.7	156.5	**0.0090**	35.4	38.8	0.2930
Afghanistan ATA	65.8	39.8	**0.0202**	31.3	54.1	0.0209
DRC Katanga	140.2	125.5	**0.0278**	89.9	116.2	**0.0280**
DRC Equateur	104.5	84.7	**0.0160**	57.2	89.8	**0.0088**
DRC Kasai	155.0	139.3	0.0749	96.4	124.3	**0.0160**
DRC CRS	46.7	46.8	0.5637	55.9	82.1	**0.0209**
Ethiopia LSTM	29.2	34.9	0.1732	68.4	133.4	**0.0088**
Ethiopia IA	44.6	50.1	0.5637	41.8	80.3	**0.0209**
Laos IOM	47.6	54.3	0.1102	61.6	71.7	**0.0433**
Laos PSI	36.9	42.7	0.1732	44.1	47.6	**0.4633**
Lesotho FIND	161.4	140.3	**0.0209**	150.5	156.1	0.7730
Nepal FHI	737.0	797.2	0.3870	93.6	92.8	0.5640
Nigeria CRS	42.4	31.5	0.0510	39.9	54.8	**0.0370**
Pakistan NTP	62.9	72.9	0.0833	40.6	91.6	**0.0209**
Pakistan IND	21.2	18.0	0.3094	43.2	72.4	**0.0209**
Rwanda WVC	41.9	44.6	0.7533	51.0	47.2	0.4633
Tanzania NIMR	7.2	5.8	0.2482	64.3	90.5	**0.0209**
Uganda BRAC	72.5	117.0	**0.0209**	79.0	100.3	**0.0209**
Uganda AMREF	79.5	74.4	0.4620	73.9	85.1	**0.0339**
Benin NTP	NA			39.6	44.7	**0.0209**
Kenya IMC	NA			189.4	176.5	0.5637
Kenya KAPTLD	NA			163.9	170.4	0.3410
Pakistan BC	NA			598.3	2278.9	0.1573
Pakistan PP	NA			53.0	171.5	**0.0209**
Somalia WVC	NA			31.8	39.8	0.2482
Sudan EPILAB	NA			136.0	132.5	0.7728
Zambia CRDRZ	NA			345.5	1500.0	**0.0433**
Zimbabwe CHD	NA			142.7	152.1	0.2482
Burkina Faso PAMAC	NA			NA		
Yemen LSTM	NA			NA		
**Totals**	**85.6**	**83.2**	**0.2482**	**69.1**	**86.2**	**0.0209**

† Sputum Smear Positive abbreviated to SS+.

Table 5 shows that no significant differences were observed in quarterly notification rate changes when stratifying projects by the presence and absence of individual case-finding activities. Although projects with improved diagnostics showed the most dramatic increases in notification rates among all case finding activities, this finding was not significant.

**Table 5:** Change in Notification Rate by Case-Finding Activity.

Case-Finding Activity	N	Median Notification Rate Change	(95% CI)	Mann-Whitney test P value
Community health workers	14	18.1	(6.4–28.7)	0.6250
No community health workers	12	9.6	(3.4–28.9)	
				
New diagnostics	7	29.2	(5.1–65.0)	0.0789
No new diagnostics	19	10.1	(4.5–24.7)	
				
Mobile outreach	15	22.8	(3.7–28.6)	0.6970
No mobile outreach	11	11.2	(5.0–46.1)	
				
Sputum transport	11	26.3	(8.3–34.3)	0.1390
No sputum transport	15	8.0	(3.4–25.6)	
				
PPM	6	20.55	(3.8–48.82)	0.5227
No PPM	20	10.65	(5.16–26.29)	
				
ACSM/Demand Generation	18	23.8	(6.9–28.8)	0.1648
No ACSM/demand generation	8	7.3	(−3.6–53.9)	
				
Contact Investigation	19	18.1	(4.4–27.4)	0.9778
No Contact Investigation	7	9.8	(2.3–72.9)	
				
Refugee/IDP/Migrants	6	5.7	(−3.2–21.5)	0.0592
No refugee/IDP/migrants	20	23.8	(6.8–29.1)	
				
Urban Slums	5	9.4	(−12.9–29.2)	0.6027
No urban slums	21	14.9	(5.3–27.2)	
				
PLHIV	5	6.5	(−0.8–11.2)	0.1109
No PLHIV	21	22.8	(6.7–28.6)	
				
Prisons	9	21.3	(−0.5–26.2)	0.6860
No prisons	17	11.2	(5.6–29.2)	
				
Other	10	11.5	(−2.6–27.4)	0.3563
No Other	16	16.3	(6.1–33.7)	

CI = Confidence Interval.

Excludes Pakistan Bridge and Zambia CIDRZ as both projects notably skew the results.

When analyses included Zambia CIDRZ and Pakistan Bridge, no significant differences were found.

**Figure 1:**
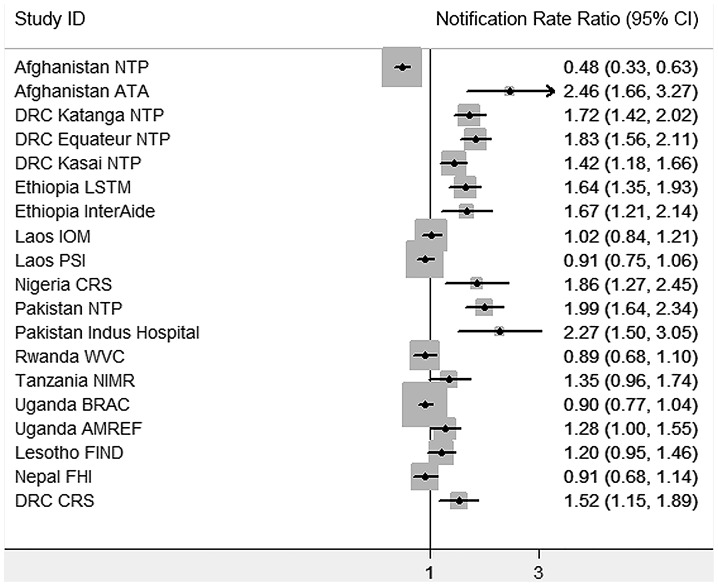
TB REACH Wave 1 forest plot of the notification rate ratios for projects with control populations.

Figure 1 shows the results of the 19 TB case finding interventions that had a control population and historical notification data. The projects' individual notification rate ratios ranged from 0.48 to 2.46, with 14 (74%) projects demonstrating increases in SS+TB notification rates in the evaluation populations from the baseline to intervention period while controlling for both historical trend and notifications in the control populations. Eleven projects had statistically significant increases in notification, while one project (Afghanistan NTP) showed a significant decrease due to an 80% rise in notification in the control population. A pooled notification rate ratio is not reported due to substantial statistical heterogeneity.

Overall, the 28 projects spent a total of 14.9 million USD for intervention activities to diagnose 17,236 additional SS+ cases.

## Discussion

The results from the 28 case detection projects show a diversity of interventions in a variety of settings with an overall large increase in SS+TB case finding, notification and treatment initiation. The gains were not explained by historical or contemporary trends, results were basically unchanged by adjustment for these factors, and no significant changes were observed in pre-selected control populations. Increased case detection was realized over a short time and included increases in case detection of SS- and extrapulmonary TB, showing that changes were not simply due to better diagnostic characterization of SS+TB. Among the projects with control populations, a 36.9% increase in SS+ case notification rates from a total population of over 60 million people was reported over baseline while in control populations there was a 3.6% decrease. The heterogeneity of individual projects' approaches and findings limits the generalizability of our results; however, the majority of interventions achieved substantial increases, suggesting that large scale active case finding interventions have high potential to improve a lagging global indicator. In order to reach the large numbers of people who remain untreated, substantial efforts are needed. Results from previous multi-county initiatives were not adjusted for historical trend or control populations [12]. Our results are consistent with prevalence surveys and other studies that have documented a high prevalence of undiagnosed TB in different populations [11], [14]–[18] and provide further support for a proactive approach to providing early diagnosis. In many active case finding publications what is described is direct yield and not additional cases [11], [14], [19]–[21]. We consider our approach to be a substantial improvement over measuring direct yield. Measuring direct yield alone does not highlight the additional impact of active case finding beyond what is routinely being done by the NTP, nor does it take notification trends into account.

While there was not enough data to perform a proper cost-effectiveness analysis, there was substantial variation in expenditure and we recognize that operating costs and efforts required to reach the people with poor access vary greatly across countries. Recently, an active case finding intervention in South Africa determined the cost to be 1,117 USD to put a person on TB treatment [20]. A review of 80 years of active TB case finding approaches noted that none followed established guidelines for cost effectiveness which future work should address [22].

Not all projects succeeded in notifying additional cases, thereby providing other lessons: projects in Kenya, Zimbabwe, and Nepal reported substantial direct yield (i.e. patients found by the project team) but did not demonstrate additional cases over expected notifications (Table S1). Patients may have still been diagnosed earlier than they would have in the absence of the interventions [23], potentially reducing case fatality and ongoing transmission, although this is speculative. It is also possible that interventions based on health systems strengthening and private sector engagement need longer than the specified one year to show a significant effect.

It is difficult to distinguish analytically what interventions work best given the heterogeneity of settings, approaches and results, but improved access to services may have played a strong role in increased notifications. This has been cited as a barrier and a way to improve case detection in other studies such as the large DETECTB study in Zimbabwe, which focused on facilitating access to services, and studies from Cambodia and Sudan focusing on decentralized services [15], [24], [25]. Interventions that included sputum transport, community outreach and better screening may be more likely to succeed than interventions focusing on equipment or specific groups at risk of TB. These vulnerable populations will vary by setting, rendering one-size fits all interventions unlikely to succeed. New diagnostics improve diagnostic certainty and may increase bacteriologically confirmed case finding [26], but we found no significant increase in median notification rates when compared to projects without new diagnostics. These data come from programmatic settings, so projects usually implemented several case finding activities rather than a single activity under controlled conditions. As a result of this, while positive project outcomes were observed, it is difficult to definitively link the success of a project to one of its several case finding activities. Future analysis will be required to more clearly identify the impact of different components on additional case notifications. Certainly approaches should be tailored to fit different epidemiological situations and country settings as with the “know your epidemic” approach used in HIV [27]. Rather than choosing from a limited set of standard options, more innovative choices should be encouraged [28].

TB REACH funding fills an important gap as major donors such as The Global Fund will not support new unproven and untested interventions. Conversely, these projects were funded nine months after the call for applications, with activities starting in less than a year. A number of the interventions have since been included in PEPFAR and The Global Fund plans based on promising outcomes (File S1).

Limitations include the marked heterogeneity of the projects with respect to design, location and results, suggesting the need for multi-site studies investigating the reproducibility of the more promising approaches before these can be more generally recommended. We did not measure diagnostic delay due to the difficulties with this estimation, but will attempt to do so in future. We have not evaluated long term trends (where an impact on TB epidemiology would be expected to lead to declining TB incidence), as this requires a much longer period of intervention, nor were projects required to estimate the impact on prevalence of undiagnosed disease, due to the high costs and logistical difficulty of this type of evaluation. Other studies [15], [29] and modeling [30]–[32] suggest active case finding can reduce TB prevalence. Finally the effect of increased burden of case notifications on treatment outcomes was not routinely measured because of the time lag involved in collecting these data, but a number of the projects improved treatment outcomes as part of the interventions (File S1) [33], [34]. Another projects' treatment outcomes were similar to those of passively found cases [35], supporting a recent systematic review which found no difference in treatment outcomes between actively and passively found cases [36]. Strengths of the evaluation include the use of official NTP data to assess additionality and judge progress, reducing the potential for project teams to over-report success, and the independent M&E team to verify project data. However, timely reporting of NTP notification data using a case-based electronic system would greatly improve data reliability and help to evaluate the impact of future case finding interventions. Reported figures are limited to individuals enrolled in treatment and so do not include cases lost before treatment or “initial default”.

## Conclusions

In summary, we have shown that large gains in TB case notification can still be achieved 20 years after the start of DOTS expansion, and at a time when global case notification trends are stagnant. Our data show the high potential of this type of fast-track funding mechanism to promote and support innovation in TB control across different settings. Independent assessment of results was a key factor that has allowed clear interpretation and avoidance of over-optimistic evaluation. These results add to the growing evidence base showing how targeted approaches to TB case finding can have a significant improvement on TB notifications [15], [16], [19], [37]. Some of the projects had negative results, showing that caution is needed in the choice of interventions, that generalization between different settings cannot be assumed, and that impact evaluation of the type described here is an essential part of all new case finding initiatives. Many people with TB across a variety of settings are still not being reached using current approaches: we propose TB REACH as a model for developing much needed innovation that can produce affordable rapid gains in efforts to control a leading global cause of morbidity and mortality.

## Supporting Information

Table S1 **Main Case Finding Strategies and Data on Direct Yield.** This table provides the main interventions in each project and a sense of the scale of direct yield of cases identified by each project. The direct yield of SS+ cases is the number of cases recorded in the project's internal monitoring as having been registered for treatment by the project as a direct result of an intervention.
(DOCX)

File S1 **Wave 1 Project Summaries.** The file contains short summaries of project approaches and a description of some of the experiences of each project to help the reader understand what was done.
(DOCX)
