# Supplementary material for: A Multi-Site Evaluation of Innovative Approaches to Increase Tuberculosis Case Notification: Summary Results
Source: PLoS One. 2014 Apr 10;9(4):e94465. doi: 10.1371/journal.pone.0094465 (PMC3983196; doi:10.1371/journal.pone.0094465)
Supplement: Table S1 — Main Case Finding Strategies and Data on Direct Yield. This table provides the main interventions in each project and a sense of the scale of direct yield of cases identified by each project. The direct yield of SS+ cases is the number of cases recorded in the project's internal monitoring as having been registered for treatment by the project as a direct result of an intervention. (DOCX) [file pone.0094465.s001.docx]

**Table S1.** Main Case Finding Strategies and Data on Direct Yield

This table provides the main interventions in each project and a sense of the scale of direct yield of cases identified by each project. The direct yield of SS+ cases is the number of cases recorded in the project’s internal monitoring as having been registered for treatment by the project as a direct result of an intervention. Some interventions therefore do not lend themselves to contribute to direct yield (advertising campaigns, laboratory strengthening, referrals etc.). The data are incomplete for some projects and all of the yields given here are not always from the same period as used to calculate additional cases.

**Main case-finding strategies and data on yield by project for TB REACH Wave 1 Projects**

| Project | Main case finding strategy/ies | Yield SS+ |
| --- | --- | --- |
| Afghanistan – NTP | 1. ACF among household contacts | 210 |
|  | 2.  ACF among prisoners | 38 |
|  | 3.  ACF in IDPs camps | 76 |
|  | 4.  Strengthening suspect management among health facilities’ clients (adults) (Training, supervision and incentives) | 4488 |
| Afghanistan - | 1.  ACF among household contacts | 136 |
| ATAAP | 2.  ACF among prisoners | 5 |
|  | 3.  ACF among IDPs/returnees | 358 |
|  | 4. Strengthening suspect management among health facilities’ clients (adults) (Training, supervision and incentives) | 1986 |
| Benin - NTP | 1.  Community-based case finding (mobile outreach teams) combined with active sensitization and mobilisation |  |
|  |  | 121 |
| DRC CRS S. Kivu | 1.   Mobile outreach teams for community-based screening (incl mobilization) | 461 |
|  | 2.   ACF among household contacts | (incl in #4) |
|  | 3.   ACF among higher risk groups (miners, prisoners, brick makers, uniformed forces) | (incl in #1) |
|  | 4a.    PPM (referral by traditional healers, club Damien) | 52 |
|  | 4a.    PPM (referral by private practitioners) | 29 |
|  | 5. Involve CODESA members and prayer rooms in sensitization and suspect identification and referral. | 521 |
|  | 6. HSS (Lab, TB suspect management among health facilities’ clients) |  |
| DRC Equateur | 1.  Community based screening and sample transport | 484 |
|  | 2.   Mobile outreach teams | 286 |
|  | 3.   ACF among household contacts | 322 |
|  | 4.   ACF among minority population | 72 |
| DRC Kasai | 1. Community based screening and sample transport | 897 |
|  | 2.  Mobile outreach teams (military and miners) | 19 |
|  | 3.  ACF among household contacts | 300 |
|  | 4.  ACF among minority populations (postponed) | n.a. |
| DRC Katanga | 1.  Community based screening and sample transport | 229 |
|  | 2.  Mobile outreach teams to minority populations | 693 |
|  | 3.  ACF among household contacts | 127 |
| Ethiopia - LSTM | 1.  Community-based case finding (health extension workers) | 2262 |
|  | 2.  ACF among household contacts | 62 |
|  | 3.  Health System Strengthening |  |
| Ethiopia- InterAide | 1.  Community-based case finding (health extension workers) | 715 |
|  | 2.  ACF among household contacts | 11 |
|  | 3.  Health System Strengthening |  |
| Kenya - IMC | 1.  Community-based case finding (community health volunteers) | 146 |
|  | 2.  Mobile outreach teams | 73 |
|  | 3.  Health System Strengthening |  |
|  | 4.  ACF among household contacts | 3 |
| Kenya - KAPTLD | 1.  Community-based case finding (screening and referral through door-to-door visits by community health workers) Fast track access at diagnostic facility | 1468 |
|  | 2. Chest camps by mobile outreach teams | 31 ( 1Ḁ398(*)1ἀ60(* |
|  | 3. ICF in HIV through peer screening | 525 |
|  | 4.  Household contact investigation | 86 |
|  | 5.  PPM activities (slum care providers in 4 slums) | 312 |
|  | 6.  PAL in 2 slums was planned but not implemented | 0 |
| Laos IOM | 1.  D2D screening and sputum collection in communities with difficult access (large proportion migrants and ethnic minorities) | 176 |
|  | 2.  Strengthening of laboratories in diagnostic centers |  |
| Laos PSI | 1. PPM (engage private providers through franchising) | 104 |
|  | 2. ACF VCT attendants |  |
|  | 3. ACF among sex workers and MSM and TG |  |
|  | 4. ACF among household contacts (started Q6 only) |  |
| Lesotho - FIND | 1.  Community-based (village health workers) and health center-based case finding | 1519 |
|  | 2.  Horse rider sputum transport service, |  |
|  | 3.  SMS text message-based system for registering TB suspects, reporting results and patient support. |  |
| Nepal - FHIl | 1. ACF among seasonal labour migrants returning from India | 302 |
|  | 2. ACF (screening and sputum collection) amongst those waiting in general and specialist OPD clinics at two public hospitals | 353 |
|  | 3. Contact Tracing in the Community | 68 |
|  | 4. ACF in HIV high risk groups at established FHI clinics (incl IDU and FSW) and others | 148 |
|  | 5. Trial of ACF at a prison and amongst street children | 1 |
| Nigeria CRS | 1.  Community based screening |  |
|  | 2.  PPM |  |
|  | 3.  ACF among clinical risk groups (diabetes, HIV positive, smokers, TB HH contacts) |  |
|  | 4. TB triage officers at all OPDs |  |
|  | 5. Sputum collection spots at remote clinics run by CVs |  |
| Pakistan - Bridge | 1.  ACF among household contacts | 489 |
| Pakistan - Indus | 1. Direct link with private GPs to screen attenders, send sputum, treat patients through incentivised GP based CHWs | 418 |
|  | 2. Advertising campaign aimed at increasing self-referral to Indus Hospital TB OPD |  |
|  | 3. Community Outreach (Chest camps): Non-HH contacts, poor neighbourhood screening | 26 |
|  | 4. Contact Tracing in the Community | 4 |
| Pakistan NTP | 1.  Mobile outreach |  |
|  | 2.  PPM |  |
|  | 3.  ACF mong FSW |  |
| Pakistan Punjab | 1.  ACF among prisoners | 340 |
| Rwanda - WVC | 1.  Health System Strengthening |  |
|  | 2.  ACF among household contacts | 36 |
|  | 3.  Community-based case finding (house to house screening and referral) | 151 |
|  | 4.  Mobile outreach team | 6 |
|  | 5.  ACF among students | 0 |
| Somalia | 1.  Health System Strengthening |  |
|  | 2.  Mobile outreach team to rural communities | 146 |
|  | 3.  ACF in higher risk groups (prisoners, IDP’s, schools, uniformed staff) |  |
|  | 4.  ACF among household contacts |  |
| Sudan - Epi - Lab | 1.  ACF in IDPs camps |  |
|  | 2.  ACF among prisoners |  |
|  | 3.  Intensified case finding among women |  |
| Tanzania - NIMR | 1.  Introducing GenXpert and LED microscopy | 357 |
|  | 2.  Mobile lab for community-based case finding | 53 |
|  | 3. Awareness raising and mobilization |  |
|  | 4.  ACF among prisoners | 196 |
| Uganda AMREF | 1. ACF among household contacts | 287 |
|  | 2. ACF among PLWHA | 840 |
| Uganda - BRAC | 1.  Community-based case finding and diagnosis (community health workers and community health volunteers) and awareness raising | 844 |
|  | 2. Increase number of diagnostic facilities by 3 |  |
|  | 3.  ACF among household contacts | 32 |
|  | 4.  ACF among prisoners (minor activity) | 4 |
| Yemen | 1.  ACF among household contacts | 152 |
| Zambia | 1.  ACF among prisoners | 165 |
| Zimbabwe | 1.  ACF among PLWHA | 3981ἀ60(*) 1  Yield SS+ |
|  | 2. HSS |  |
|  | 3. Community screening and sputum collection points | 248 |
|  | 4.  ACF among household contacts | 60 1  Yield SS+ |
