# Supplementary material for: A Multi-Site Evaluation of Innovative Approaches to Increase Tuberculosis Case Notification: Summary Results
Source: PLoS One. 2014 Apr 10;9(4):e94465. doi: 10.1371/journal.pone.0094465 (PMC3983196; doi:10.1371/journal.pone.0094465)
Supplement: File S1 — Wave 1 Project Summaries. The file contains short summaries of project approaches and a description of some of the experiences of each project to help the reader understand what was done. (DOCX) [file pone.0094465.s002.docx]

**File S1.** Wave 1 Project Summaries

Anti-TB Association Afghanistan Program (ATA-AP) – Domestic NGO.

Systematic screening of all attendees at government facilities was conducted and financial incentives were offered for smear positive cases found for the facility. In addition, training and supervision in suspect management was provided. Mobile units were deployed for systematic screening of people living in internally displaced person camps, and TB household contact investigation was conducted. Case finding activities were conducted in prisons which involved screening inmates as one-off events. The evaluation population as stated comprised 47 health facilities plus some mobile clinics, therefore possible 'redistribution' of cases in the same BMUs was possible. A wider analysis of provincial data from Afghanistan showed large increases overall in the project area, so this concern was alleviated. Continuation of active case finding among IDPs is now supported by JICA. Project was continued in year 2 by TB REACH.

Afghanistan NTP - National TB Program

Afghanistan’s NTP implemented a program in 10 provinces to increase TB case detection among internally displaced persons/returnees, prisoners, household contacts of SS+ cases, and those who previously attempted to seek TB services. Visiting health workers went door to door to increase awareness of TB diagnosis and treatment. Improved management of people with suspected TB among health facilities was prioritized through training and an incentive program. Because of a clear seasonality pattern in the Afghanistan NTP project and a five-quarter implementation period, the trend adjusted baseline was calculated slightly different: instead of the intercept the notification one year prior to the quarter was used to predict notification (e.g. for Q4/10 the prediction was calculated as follows: Q4/09 + (4*α).

Benin NTP - National TB Program

A total of 120 communities were targeted with promotion of TB awareness and information on TB detection and free treatment. Mobile units deployed to the areas and worked with village leaders to provide community TB education. The mobile teams used LED fluorescent microscopy to diagnose sputum from people with suspected TB. Those found to be smear-positive were subsequently linked to the NTP to receive free treatment.

Burkina Faso NTP - National TB Program

This project was excluded from analysis because case finding activities began after December 31^st^, 2011.

DR Congo Catholic Relief Services (CRS) - International NGO

CRS implemented an intervention that strengthened coordination between the Provincial Tuberculosis Program, private practitioners and traditional healers. In addition, nurses, lab technicians and community health volunteers forming mobile outreach teams provided education, diagnosis and treatment services to high risk groups including prisoners, miners, brick makers, uniformed forces, and TB household contacts. CRS also worked with local organizations to identify and refer suspects from prayer rooms, and worked on health system strengthening by providing lab support and improved TB suspect management. Project was continued in year 2 by TB REACH.

DR Congo Katanga – NTP - National TB Program

In Katanga province, the NTP conducted community based screening and sample transport by mobilizing former TB patients and trained community volunteers to actively screen TB contacts, and provided outreach to isolated ethnic groups in satellite health centers located in communities with low detection rates. Former TB patients visited primary schools to teach children how to recognize and respond to TB, and radio broadcasts were used to inform people about the signs, symptoms and treatment options for TB. Parts of project was continued in year 2 by TB REACH.

DR Congo South Equator – NTP - National TB Program

In DRC’s Equator Province, in areas where marginalized pygmies have limited access to healthcare, trained community volunteers traveled to these groups as well as their household contacts to help identify people exhibiting signs of TB. Sputum samples were transported via bicycle or canoe for diagnosis, and then TB positive patients were initiated on treatment. Local traditional and religious leaders, and administrative bodies provided support to help gain community acceptance. Parts of project was continued in year 2 by TB REACH.

DR Congo West Kasai – NTP - National TB Program

After the NTLP identified the main barriers to accessing TB diagnosis and treatment, they enlisted former TB patient volunteers to identify and refer TB suspects among military personnel, miners and household contacts, and to generate greater community awareness around TB. The TB patient volunteers subsequently transported sputum back to labs for diagnosis. There was some civil unrest in some project areas during the implementation that may have disturbed some of the case finding activities but it was not this was not quantified. Parts of project was continued in year 2 by TB REACH.

Ethiopia Inter Aide - International NGO

The project focused on a few main operations in certain areas of Dawro Zone. Setup a field-based referral system: mobilize and train Health Extension Workers (HEW) and Community Volunteers (CV) to pro-actively inform, identify and refer TB suspects through regular screening of the population, including on-site sputum collection for isolated areas. Increase performance and capacity of diagnostic units to deliver in and out patient quality services to care seekers. Organise training for lab professionals, provide material support, and assist the local health office in improving access to quality diagnostic and determining hotspots for outreach sputum collection. Sensitize top health professionals about the existing challenges regarding TB control. Project was continued in year 2 by TB REACH.

Ethiopia London School of Tropical Medicine - Foreign Academic Institution

Conducted in Ethiopia’s Sidama Zone, the main interventions of the project include conducting extensive advocacy, communication and social mobilization, engaging all stakeholders, the communities and the Health Extension workers (HEWs) in TB control activities, training of HEWs and staff working in the TB clinics. The project distributed LED-FM microscopes to improve laboratory capacity. HEWs conducted house-to-house visits to identify TB suspects, collect sputum samples, prepare and fix smears within their community and contact supervisors by mobile phone. Supervisors are employed by the project and travel to the villages by motorbikes to collect and transport smeared slides for microscopy in the laboratories, initiate treatment for smear-positive TB cases and screen their household contacts and initiate Isoniazid preventive therapy for asymptomatic young children. HEWs support and supervise treatment and report outcome. Once patients were positively identified, they were initiated on treatment and aided in further contact screening with the support of the Southern Region Health Bureau and Sidama Zone Health Department. Treatment outcomes from the cohorts in intervention areas showed 93% treatment success compared to 77% in the baseline period. Project was continued in year 2 by TB REACH and plans to link with Global Fund support are being considered.

Kenya International Medical Corps (IMC) - International NGO

The project trained 120 health care providers and 172 community health workers on TB detection and treatment, facilitated refresher training for 22 public health facility members, refreshed 90 laboratory staff on AFB microscopy, and established 9 new TB diagnostic sites across the districts implementation. Active TB case finding was carried out through mobile outreaches, door to door, moonlight, contact tracing and screening of HIV clients for TB. The project worked with local drug sellers who offered their shops as sputum collection centers for TB testing, assisted in screening clients, as well as in disseminating information regarding TB and its treatment. The community volunteers followed up with patients regarding their treatment regimen and clinic appointments in order to ensure adherence. Most activities have been integrated into routine practice in the health facilities supported by the project.

Kenya KAPTLD - Domestic NGO

KAPTLD mobilized former and current TB/HIV patients to work as peer screeners of TB among people living with HIV. Also, community health workers held screening camps and carried out door to door screening campaigns, while chemists/shopkeepers offered their shops as fast-track access points to identify and TB suspects, reducing the traditional 6-12 week diagnosis delay to less than one month when measured by project internal monitoring. Project was continued in year 2 by TB REACH.

Laos International Organization for Migration (IOM) International Organization

IOM and National TB Centre conducted door to door screening of migrants and ethnic minorities, and identified TB suspects among them living in and crossing through rural provinces Savannakhet and Champasakby. Informational campaigns and modern training for laboratory technicians were also implemented, as well as mass screenings and sputum collection and transport, by trained village health volunteers.

Lao PSI - International NGO

Population Services International together with Lao’s National Tuberculosis Centre created a network of private clinics called Sun Quality Health, that offered free and high quality TB diagnosis and treatment as part of a public-private partnership. To improve numbers of people screened, SMS and Google Earth technology were used to send educational messages and Sun Quality Health referrals to people identified as belonging to high-risk populations, including VCT attendants, sex workers, MSM, Transgenders, and household contacts. An expansion of the project was included in a successful Global Fund application ensuring continued implementation.

Lesotho FIND - International NGO

FIND and Lesotho’s Ministry of Health and Social Welfare increased TB case detection in more than 120 remote communities, whose main or only options for transport are walking or horseback riding. Teams of hired horse riders traveled to intervention communities 2 to 3 times per week to pick up sputum samples and deliver them to health centres, after village health workers had screened community members suspected of having TB. SMS technology was then used to communicate patients’ results to these health workers, who then immediately started and ensured treatment of patients in their communities. The SMS technology was also used to track TB and MDR-TB patient progress, and in generating monthly reports. There was a large, unexplained spike in notification in Q4/10 during the baseline period which makes the additional case calculation appear lower than it probably was. Smoothing out the line over the baseline period for a possible accumulation of registered cases was considered but ultimately we analyzed the data as it was reported by the NTP. Project was continued in year 2 by TB REACH.

Nepal FHI - International NGO

The project screened OPD attendees, migrant workers, injection drug users and sex workers, as well as prisoners and street children for TB because of their limited access to and use of health services in Rupandehi district bordering northern India. Contact investigation was also carried out in these communities to find more people with TB symptoms. The project then offered these groups as well as their contacts sputum testing, treatment if necessary, and follow-up for treatment compliance. In Nepal year is divided in 3 periods of 4 months (Nov-Feb=T2; Mar-Jun=T3; Jul-Oct=T1). We converted the data to 3 month quarters to standardize reporting.

Nigeria CRS - International NGO

Centered in Kogi and Enugu states in Nigeria, the project trained 120 community workers and volunteers on TB case detection and follow up; integrating cough triage officers in all out-patient departments (OPD) of the 18 target facilities. The project conducted promoted screening all clinical risk groups for TB with focus on HIV positives, diabetics, smokers, family members of TB suspects and previous TB patients. systematically screening household contacts of smear positive TB cases. The intervention strengthened 18 TB microscopy laboratories in smear diagnosis and provided quarterly technical assistance visits. A number of other trainings were held for health facility staff and work on establishing innovative community-based systems for sputum collection with linkages to TB diagnosis centres was conducted. Community volunteers also collected and transported sputum from remote clinics. There was a strong outreach and communications strategy in the communities and an aspect on ensuring adherence to treatment for cases detected but no data was provided on this measure.

Pakistan Bridge Consultants – Domestic NGO

Pakistan Bridge Consultants and Ojha Institute of Chest Diseases implemented case finding activities among TB household contacts in 5 urban and 5 rural districts. Active case finding involving visiting TB patients’ homes (more than once if required) by trained community mobilizers were able to identify contacts, and to bring people with suspected TB to local health centers for sputum microscopy where it was collected in a frontloaded manner, ensuring no repeat visits to the health center. The social mobilizers then followed up patients identified as SS+ throughout the treatment period. An incentive of a monthly food basket was also given to patients and treatment success was 97% for the contacts identified with SS+ TB with no treatment interruptions whereas the rate in Sindh province is 6% overall. Because this project targeted TB patient contacts, no suitable evaluation population could be determined, so the number of patients initiating treatment who had a contact history was used. The current practice of contact investigation is a passive approach that had led to 34 cases being detected in the project area the year before. Project was continued in year 2 by TB REACH and expansion of contact investigation is included in the current Phase 2 Global Fund Grant.

Pakistan Indus Hospital - Domestic NGO

In Karachi, Pakistan, the Indus Hospital adopted a multi-pronged approach to improve case detection and case holding for TB. The approach included partnerships with private health clinics, contact tracing amongst household contacts of TB patients, active case finding in vulnerable communities, and mass communications campaign designed to increase self-referral of symptomatic suspects to the Indus Hospital and its partner clinics. Community mobilizers were remunerated through performance-based incentives for form filling, sputum collection, case detection, and follow-up. Please refer to reference (34) for a full description of the project and outcomes. Project was continued in year 2 by TB REACH and expansion of PPM activities is included in the current Phase 2 Global Fund Grant.

Pakistan NTP - National Tuberculosis Program

Mobile outreach teams were deployed daily in different chest camps to increase TB case detection in urban slum areas in Sindh province that were in partnership with local private providers. A local team spent time in the community before each camp promoting the event and camps screened hundreds of people. The NTP conducted community education, additional training for general practitioners in TB case management, and established chest camps that utilized active case finding, to find additional cases of TB. Project was continued in year 2 by TB REACH and expansion PPM and active case finding is included in the current Phase 2 Global Fund Grant.

Pakistan Punjab Prisons – Provincial TB Program

In this project, active case finding was conducted among prisoners through a systematic screening process at 32 prisons across Punjab Province. In many cases TB posts were set up at the prison clinic for the first time. Standard procedures and screening guidelines were developed, trainings conducted and prisons were equipped with laboratory equipment for diagnostic procedures.

Rwanda World Vision – International NGO

Implemented in four districts: Gasabo, Bugesera, Nyamagabe and Gicumbi,World Vision and Rwanda’s Ministry of Health collaborated to focus on door-to-door screening and referral by community health workers, community DOT provision, contact investigation and a large behavioral change and community education component. The project also had a large focus on system strengthening and conducted a number of trainings around DOTS and equipped laboratories with microscopes and supplies.

Somalia World Vision - International NGO

The project was implemented using a total of 6 mobile teams (3 in Puntland and 3 in Somaliland) who went to the hard to reach communities (nomads, prisoners, the military, IDPs and communities far from health facilities) and conducted active case finding and referrals for treatment. Ongoing care and treatment supervision were provided by trained community volunteers. Existing health facilities were identified for DOTs and new centres were also established to administer the drugs to outpatients and those continuing with treatment. Community DOTS persons were trained to follow up cases, administer drugs through DOTs and report progress in liaison with the project staff. The evaluation population’s case detection had been declining for the three years prior to implementation, and this remained unchanged throughout project activities, which may have been related to issues, population displacement, and political unrest. No data was available for all forms of TB, only for confirmed cases and in a couple of instances project staff could not access certain TB facilities.

Sudan Epi-Lab - Domestic NGO

Sudan Epi-Lab trained women to become peer educators of women in their social networks, in order to identify and refer additional cases of TB to address the situation that in North Kordufan and Gazira states in Northern Sudan, men are twice as likely as women to have been tested and treated for tuberculosis. For each referral made the female volunteers received a small financial incentive, and additional incentives for every patient helped through treatment adherence. These services were also provided in ten IDP camps, and among prisoners. The project had a health systems strengthening component as well which focused on military hospitals.

Tanzania Munich University - Foreign Academic Institution

In this project, the Mbeya Medical Research Centre and University of Munich introduced new diagnostic tools to the Mbyea Region. Smaller health facilities were equipped with LED microscopes, bigger facilities such as one District Hospital, one Health Centre, both border facilities, as well as one Regional Hospital in the urban area, were provided with a GeneXpert machines. The project targeted a large rural population with difficult access to health services including a mobile van with Xpert MTB/RIF testing and awareness campaigns, a prison intervention and an urban intervention where the focus was on improved services in communities with poor access to care. Due to the fact that the project was the first project using GeneXpert technology in Tanzania there were a number of reporting issues around registering cases and the impact of improved numbers of bacteriological TB may have been underestimated. Historical baseline data for all forms of TB in the evaluation population was not available. The project has secured funding for continuing activities and expansion of services from PEPFAR funds.

Uganda AMREF - International NGO

The project trained health workers to identify TB suspects in a programme called “Two Day- integrated community outreach”, in Luwero to increase access to TB treatment services to TB household contacts and PLWHA. Community health workers obtained sputum samples from TB suspects over two consecutive days, which the health workers would then transport for diagnosis to a public facility trainings and supervision were conducted on screening and laboratory diagnosis. People diagnosed with TB were supported throughout their treatment regimen with DOT. One district registered serious shortages of TB drugs which resulted in many people preferring to travel to Kampala for diagnosis and treatment, rather than seeking care within the district.

Uganda BRAC - International NGO

The project focused on community outreach, strengthening diagnostic facilities and contact investigation. Three additional diagnostic facilities were supported. Female health promoters educated community members and TB household contacts, with basic health information, products and services, including those related to TB. People with suspected TB were referred to a public diagnostic facility for testing. The health promoters were rewarded with a small incentive for all SS+ TB cases that successfully finished treatment. Project was continued in year 2 by TB REACH and approved as a sub-recipient for Global Fund by the NTP.

Yemen London School of Tropical Medicine - Foreign Academic Institution

Yemen’s National TB Institute and the Liverpool School of Tropical Medicine worked to improve TB case detection in three groups: women, children, and the elderly. This was done by health workers who collected sputum from household contacts that were identified during home visits to current TB patients. Children were referred to hospitals for a complete diagnosis because of the difficulty of obtaining pediatric sputum samples. All identified cases were started on TB treatment. This project was excluded from analysis because the organization was unable to report for the entire evaluation population due to civil unrest in the country.

Zimbabwe Harare City – Local Health Department

The Harare City Health Department decentralized TB laboratory services through sputum sample transport, and focused on systematically screening with a focus on household contacts of TB patients and HIV positive patients. The project was able to successfully add a number of microscopy centers in the Harare City area whereas prior to the project all sputum was centrally processed leading to long delays. The project also focused on improving training of health personnel. The project was unable to provide historical baseline data for all forms of TB despite visits from the M&E team. Much of the project focus on decentralization of lab services has been integrated into routine practice by the City of Harare.

Zambia CIDRZ – Domestic NGO

In Zambia’s Lusaka Central Prison, some inmates worked as TB Peer Educators, in which they collected sputum samples from screened TB suspects, which were then tested under fluorescence microscopy. In a mobile screening facility, other TB suspects were screened, counseled and tested for HIV, and then examined via digital chest x-ray. The use of digital chest x-ray allowed for images to be sent for remote reading and diagnosis, considerably cutting down time to diagnosis. The project conducted mass screening, periodic, and entry screening as well as work with the prison workers. The project did not establish a larger evaluation population, so notification data for the target population (prison only) was provided. Project continued after 30 Sept 2011, but no case finding activities were conducted. The project received funding from the European Union and US CDC for continued and expanded services.
